# Synthesis and crystal structure of poly[[μ-chlorido-μ-(2,3-di­methyl­pyrazine)-copper(I)] ethanol hemisolvate], which shows a new isomeric CuCl(2,3-di­methyl­pyrazine) network

**DOI:** 10.1107/S2056989024009174

**Published:** 2024-09-24

**Authors:** Christian Näther, Inke Jess

**Affiliations:** a Institut für Anorganische Chemie, Universität Kiel, Max-Eyth.-Str. 2, 24118 Kiel, Germany; Institute of Chemistry, Chinese Academy of Sciences

**Keywords:** synthesis, copper(I) coordination polymer, 2,3-di­methyl­pyrazine, crystal structure

## Abstract

Reaction of copper(I)chloride with 2,3-di­methyl­pyrazine in ethanol leads to the formation of the title compound, poly[[μ-chlorido-μ-(2,3-di­methyl­pyrazine)-copper(I)] ethanol hemisolvate], {[CuCl(C_6_H_8_N_2_)]·0.5C_2_H_5_OH}_*n*_ or CuCl(2,3-di­methyl­pyrazine) ethanol hemisolvate. Its asymmetric unit consists of two crystallographically independent copper cations, two chloride anions and two 2,3-di­methyl­pyrazine ligands as well as one ethanol solvate mol­ecule in general positions. The ethanol mol­ecule is disordered and was refined using a split model. The methyl H atoms of the 2,3-di­methyl­pyrazine ligands are also disordered and were refined in two orientations rotated by 60° relative to each other. In the crystal structure, each copper cation is tetra­hedrally coordinated by two N atoms of two bridging 2,3-di­methyl­pyrazine ligands and two μ-1,1-bridg­ing chloride anions. Each of the two copper cations are linked by pairs of bridging chloride anions into dinuclear units that are further linked into layers *via* bridging 2,3-di­methyl­pyrazine coligands. These layers are stacked in such a way that channels are formed in which the disordered solvent mol­ecules are located. The topology of this network is completely different from that observed in the two polymorphic modifications of CuCl(2,3-di­methyl­pyrazine) reported in the literature [Jess & Näther (2006). *Inorg. Chem.***45**, 7446–7454]. Powder X-ray diffraction measurements reveal that the title compound is unstable and transforms immediately into an unknown crystalline phase.

## Abstract 2

In the crystal structure of the title compound, the copper cations are each tetra­hedrally coordinated by two 2–3-di­methyl­pyrazine ligands and two chloride anions and linked into dinuclear units that are further connected into layers by bridging 2,3-di­methyl­pyrazine ligands.

## 1. Chemical context

Many coordination compounds based on copper(I) halides and N-donor coligands are reported in the literature and some of them are of inter­est because of their luminescence properties (Näther *et al.*, 2003 ▸; Jess *et al.*, 2007*a* ▸; Pospíšil *et al.*, 2011 ▸; Gibbons *et al.*, 2017 ▸; Mensah *et al.*, 2022 ▸). The main inter­est, however, originates from their extremely versatile structural behavior, for which there are two main explanations (Kromp & Sheldrick, 1999 ▸; Peng *et al.*, 2010 ▸; Näther & Jess, 2004 ▸; Li *et al.*, 2005 ▸). Firstly, such compounds consist of different Cu*X* substructures such as monomeric or dimeric units, rings, chains and double chains because halide anions are able to connect metal cations *via* the μ-1,1-bridging mode (Näther *et al.*, 2013 ▸). These Cu*X* substructures can be further connected if bridging coligands are used in the synthesis. Secondly, for a given copper halide and a given coligand, frequently several compounds of different stoichiometry exist in which the ratio between Cu*X* and the coligand vary (Näther *et al.*, 2013 ▸). In most cases, the compounds with a small Cu*X*:coligand ratio can easily be prepared in solution using conventional solvents or, in some cases, using the pure coligand. In contrast, the compounds with a large ratio between Cu*X* and coligand are frequently difficult to prepare in solution but are mostly accessible by thermal ligand removal starting from the colig­and-rich compounds (Näther *et al.*, 2001 ▸, 2002 ▸, 2013 ▸; Näther & Jess, 2001 ▸). In this case, compounds with more condensed Cu*X* networks will form. This procedure can be used for the synthesis of a wide range of coordination compounds with different cations and different anionic ligands such as, for example, thio- and seleno­cyanates (Näther *et al.*, 2013 ▸).
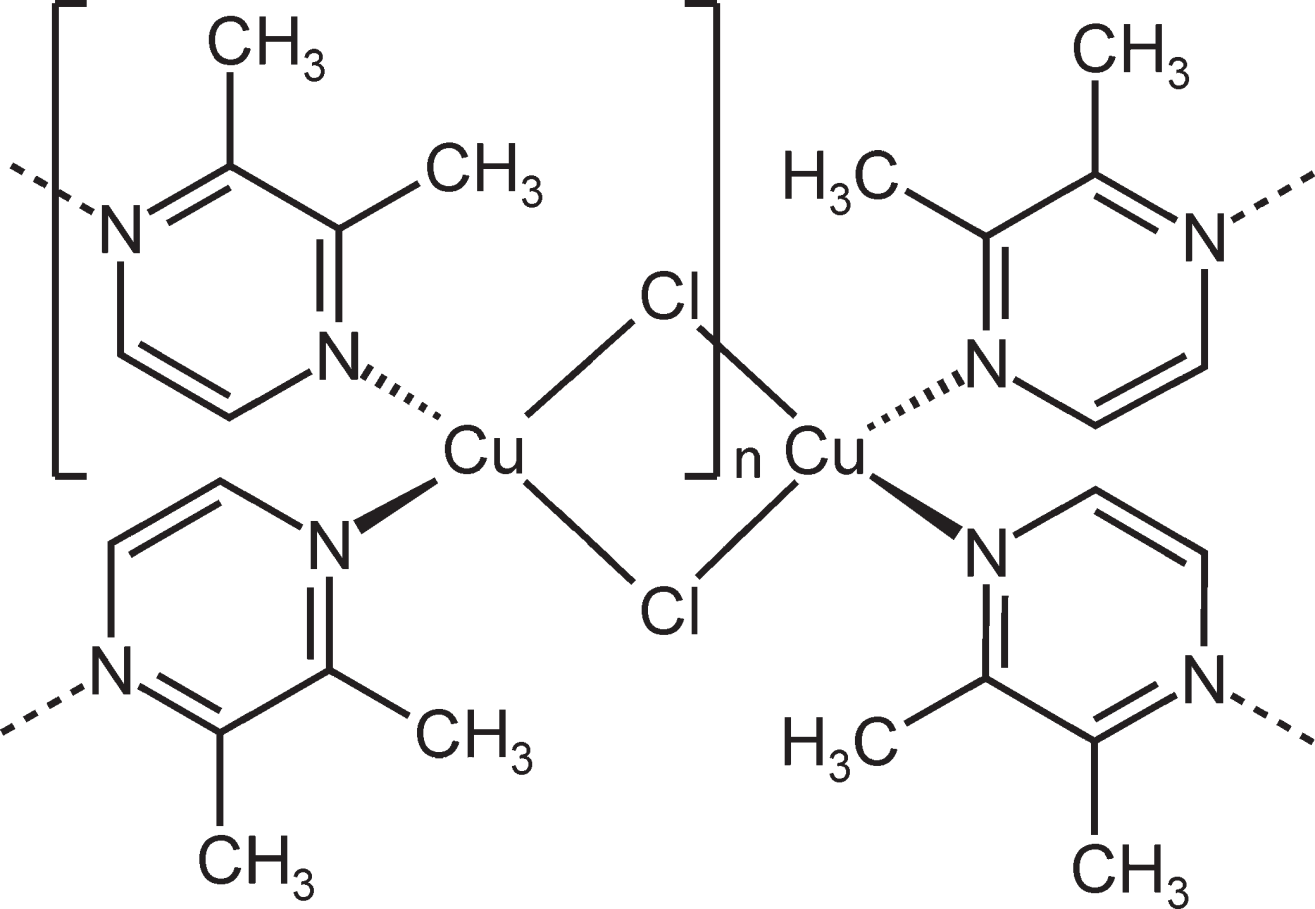


In this context we have reported on compounds based on CuCl and 2,3-di­methyl­pyrazine as coligand (Jess & Näther, 2006 ▸). In the most 2,3-di­methyl­pyrazine-rich compound, (CuCl)_4_(2,3-di­methyl­pyrazine)_6_-tris­(2,3-di­methyl­pyrazine) solvate, the copper cations are tetra­hedrally coordinated by two chloride anions and one terminal as well as one bridging 2,3-di­methyl­pyrazine coligand. Each of the two copper cations are linked by two μ-1,1-bridging chloride anions into (CuCl)_2_ rings and two such rings are linked *via* the two bridging 2,3-di­methyl­pyrazine ligands into discrete tetra­nuclear complexes. If the solvate mol­ecules are not considered, the ratio between CuCl and coligand is 1:2. If the discrete complexes are heated, some of the coligands are removed and a transformation into (CuCl)_3_(2,3-di­methyl­pyrazine)_2_ (ratio 3:2) is observed. This loses additional coligands upon further heating and finally transforms into (CuCl)_2_(2,3-di­methyl­pyrazine) with a CuCl:coligand ratio of 2:1. In the 3:2 compound, six-membered (CuCl)_3_ rings are observed, which are connected by the 2,3-di­methyl­pyrazine ligands into chains, whereas the 2:1 compound consists of CuCl double chains that are connected by the 2,3-di­methyl­pyrazine ligands into layers. Two additional compounds with the composition CuCl(2,3-di­methyl­pyrazine) (ratio 1:1) were obtained from solution, which transform into the 3:2 compound upon heating (Jess & Näther, 2006 ▸). One of the 1:1 compounds is thermodynamically stable at room temperature and consists of dinuclear (CuCl)_2_ units that are connected by the 2,3-di­methyl­pyrazine ligands into layers. In the metastable isomer, similar (CuCl)_2_ units are observed that are linked by the coligands into layers, but the layer topology is different. Much later we accidentally found crystals of an additional compound with a ratio of 1:1 that contains ethanol as solvent and that was characterized by single crystal X-ray analysis. Investigations using powder X-ray diffraction revealed that this compound is unstable and transforms into an unknown crystalline phase upon storage and this might be the reason why it was overlooked in our previous work.

## 2. Structural commentary

**Figure 1:**
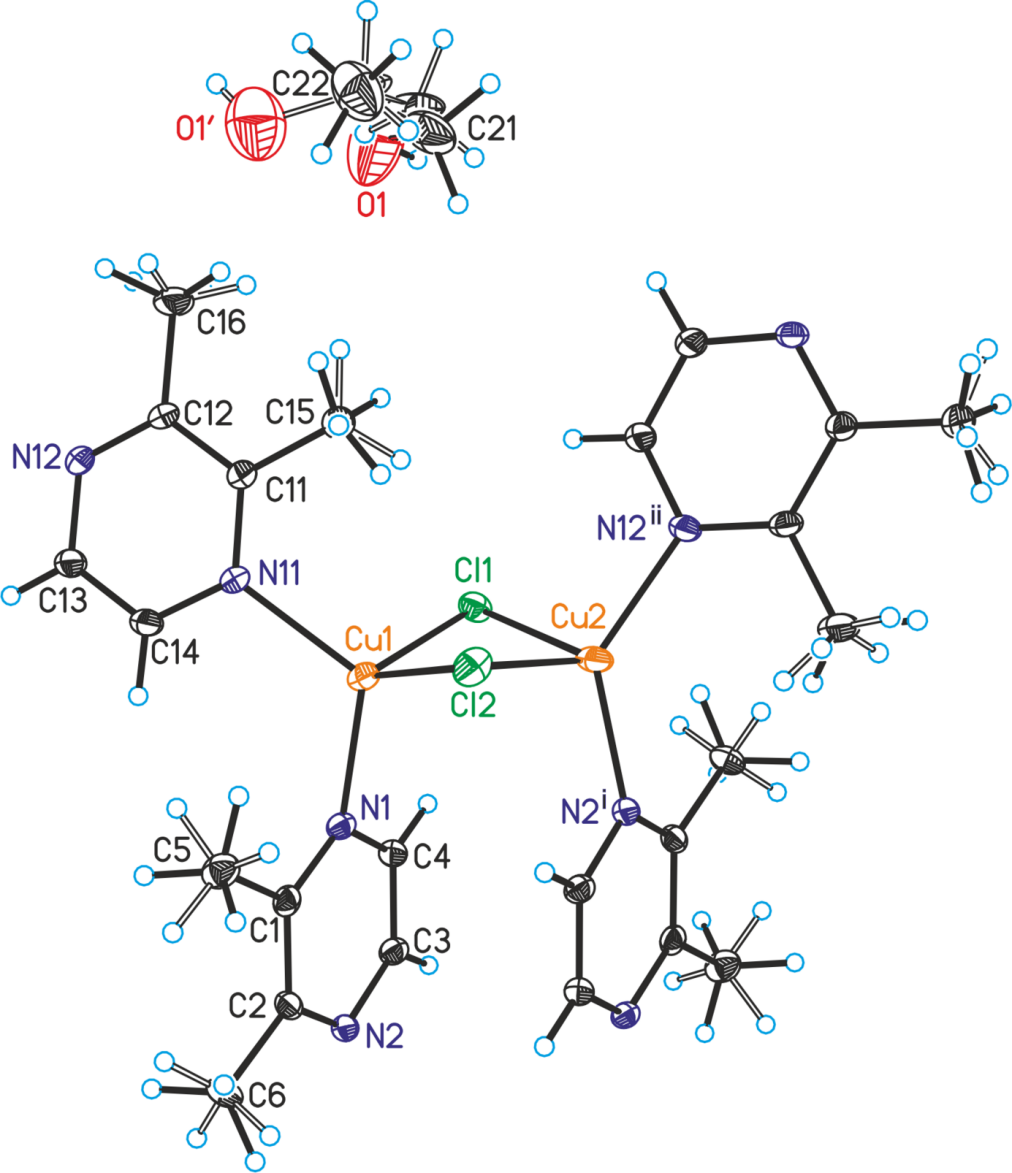
Crystal structure of the title compound with labeling and displacement ellipsoids drawn at the 50% probability level. The disorder of the methyl H atoms and of the ethanol mol­ecule is shown with full and open bonds. Symmetry codes for the generation of equivalent atoms: (i) −*x* + 2, −*y* + 1, −*z* + 1; (ii) −*x* + 1, *y* + 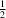, −*z* + 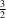.

**Figure 2:**
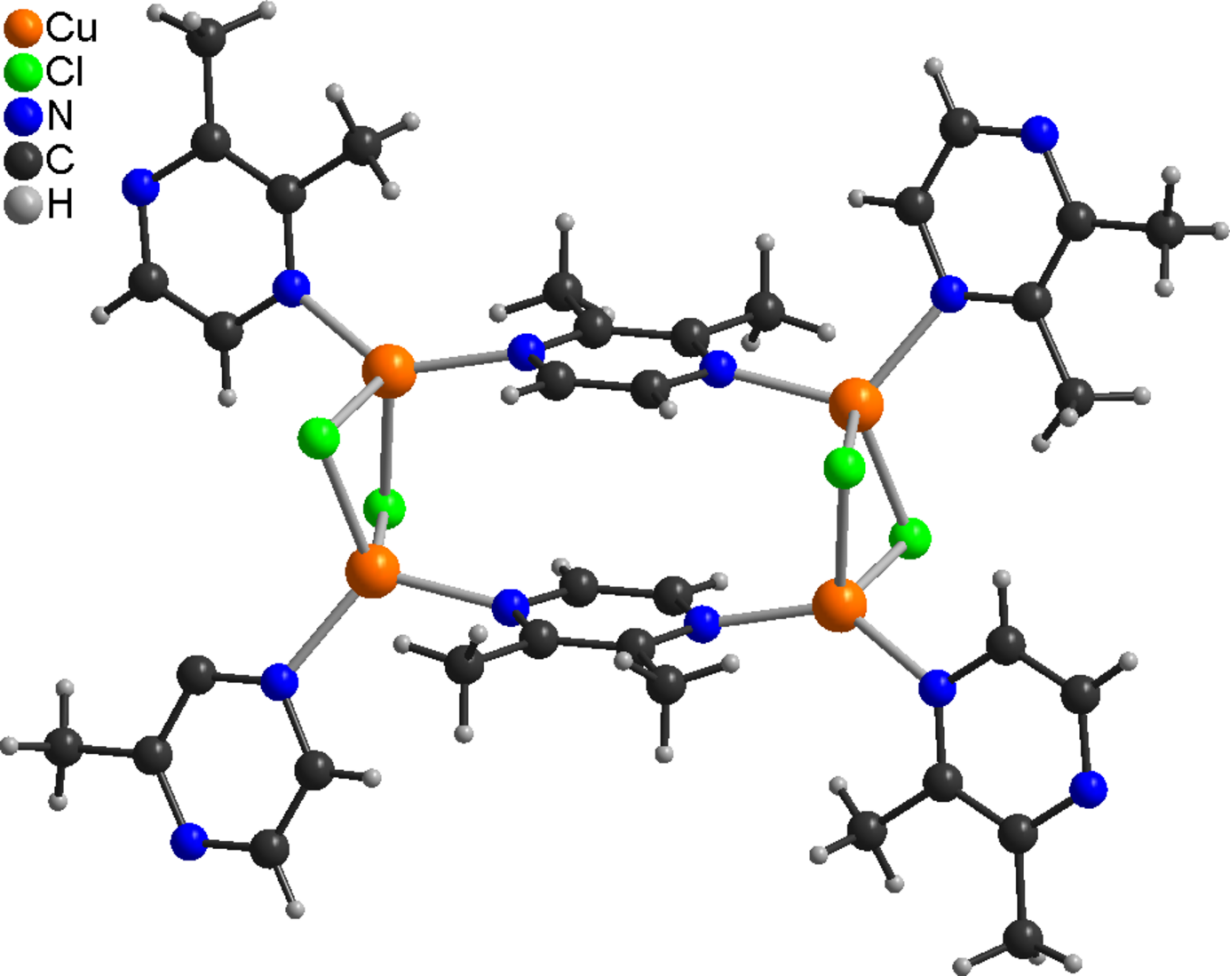
View of the(CuCl)_4_(2,3-di­methyl­pyrazine)_6_ unit.

**Figure 3:**
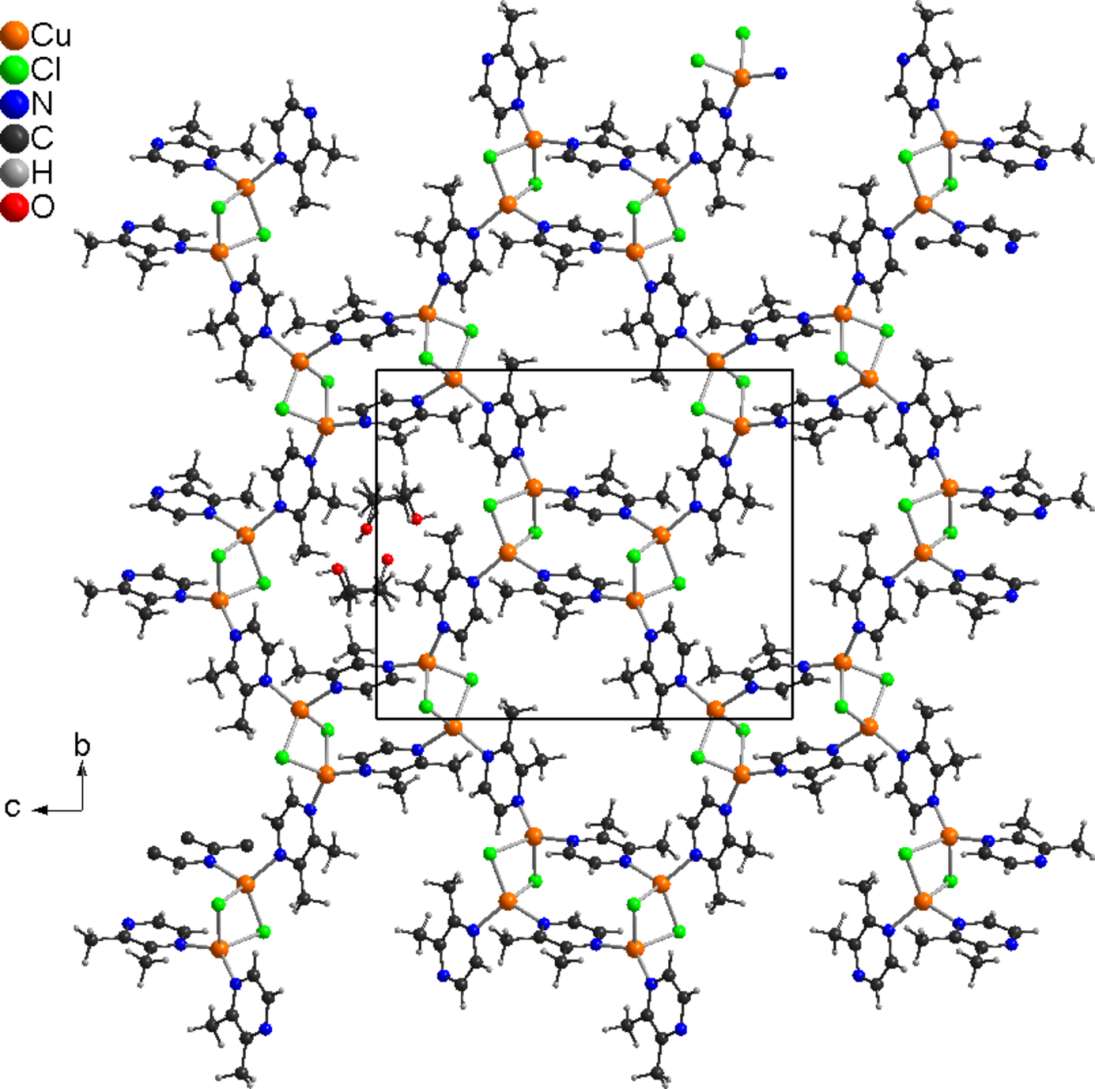
Crystal structure of the title compound in a view along the crystallographic *a* axis. Only one position for the disordered ethanol mol­ecules is shown.

The asymmetric unit of the title compound, poly[CuCl(2,3-di­methyl­pyrazine) ethanol hemisolvate], consists of two crystallographically independent copper(I) cations, chloride anions and 2,3-di­methyl­pyrazine coligands as well as of one ethanol mol­ecule in general positions (Fig. 1 ▸). The methyl H atoms of all 2,3-di­methyl­pyrazine coligands are disordered, which is also the case for the ethanol mol­ecule, which was refined using a split model. Each Cu cation is coordinated by two bridging chloride anions and two N atoms of two 2,3-di­methyl­pyrazine coligands within slightly distorted tetra­hedra (Fig. 1 ▸ and Table 1 ▸). The Cu cations are linked by the two μ-1,1-bridging chloride anions into (CuCl)_2_ units, in which the Cu⋯Cu distance is 2.9516 (5) Å (Table 1 ▸). Both of these (CuCl)_2_ units are linked by two bridging 2,3-di­methyl­pyrazine units into (CuCl)_2_(2,3-di­methyl­pyrazine)_2_ building blocks (Fig. 2 ▸) that are further connected into layers by additional bridging 2,3-di­methyl­pyrazine coligands (Fig. 3 ▸).

In this context it is noted that the title compound shows a new isomeric CuCl(2,3-di­methyl­pyrazine) network that is completely different from that reported for the two polymorphic modifications CuCl(2,3-di­methyl­pyrazine) (Jess & Näther, 2006 ▸). In both forms, (CuCl)_2_ units are observed in which the copper cations are tetra­hedrally coordinated. These units are linked by bridging 2,3-di­methyl­pyrazine ligands into larger rings built up of four (CuCl)_2_ units and four 2,3-di­methyl­pyrazine ligands that finally condense into layers (Fig. S1 in the supporting information). The topology of the network is identical in both forms, but in the ortho­rhom­bic polymorph (Fig. S1: top) the rings are perfectly stacked onto each other, which is not the case in the monoclinic form (Fig. S1: bottom).

## 3. Supra­molecular features

In the crystal structure of the title compound, the layers are stacked in such a way that cavities are formed in which the disordered ethanol mol­ecules are embedded (Fig. 3 ▸). These ethanol mol­ecules are linked by inter­molecular O—H⋯Cl and C—H⋯O inter­actions into layers parallel to (102) (Table 2 ▸). There are a number of C—H⋯Cl inter­actions, but for most of them the C—H⋯Cl angles are far from linear with large H⋯Cl distances, indicating only weak inter­actions (Table 2 ▸).

## 4. Database survey

As already mentioned above, several compounds based on CuCl and 2,3-di­methyl­pyrazine are listed in the CCDC database (CSD Version 5.43, March 2024; Groom *et al.*, 2016 ▸). These include (CuCl)_4_(2,3-di­methyl­pyrazine)_3_-tris­(2,3-di­methyl­pyrazine) solvate (Refcode JEPPAW, Jess & Näther, 2006 ▸), (CuCl)_3_(2,3-di­methyl­pyrazine)_2_ (Refcodes JEPPEA, Jess & Näther, 2006 ▸ and JEPPEA01, Turnbull *et al.*, 2020 ▸), (CuCl)_2_(2,3-di­methyl­pyrazine (Refcode JEPPIE, Jess & Näther, 2006 ▸) and two isomers of CuCl(2,3-di­methyl­pyrazine (Refcodes JESXEL and JESXEL01, Jess & Näther, 2006 ▸). There is also one compound with the composition (CuCl)4(2,3-di­methyl­pyrazine)_4_(aceto­nitrile)_4_ (Refcode KICZEC, Jess & Näther, 2007 ▸) that forms tetra­nuclear units.

It is noted that two compounds have been reported that contain copper(II) cations. In one, CuCl_2_(2,3-di­methyl­pyrazine (Refcode: GEDTOA, Jornet-Somoza *et al.*, 2012 ▸), the copper cations are fivefold coordinated by one terminal and two μ-1,1-bridging chloride anions in a trigonal–bipyramidal coordination. Each of the two copper cations are linked by pairs of μ-1,1-bridging chloride anions into Cu_2_Cl_6_ units that are further linked into double chains by bridging 2,3-di­methyl­pyrazine ligands. No atomic coordinates are given for the other compound, CuClNO_2_-(2,3-di­methyl­pyrazine) (Refcode XIGKAB, Xiao *et al.*, 2010 ▸).

Finally, some 2,3-di­methyl­pyrazine compounds with copper(I) cations and bromide as well as iodide anions are also known, including (CuBr)_2_(2,3-di­methyl­pyrazine, which is not isotypic to its Cl analog (Refcode KICZOM, Jess *et al.*, 2007*b* ▸), CuBr(2,3-di­methyl­pyrazine) (Refcode QIJTEI, Näther & Greve, 2001 ▸) and (CuBr)_3_(2,3-di­methyl­pyrazine)_2_, which is isotypic to its Cl analog (Refcode XANKIH, Wells *et al.*, 2005 ▸).

For CuI and 2,3-di­methyl­pyrazine, two compounds are listed in the CSD, *viz*. (CuI)_2_(2,3-di­methyl­pyrazine)_3_ (Refcode LIDXOM, Jess *et al.*, 2007*a* ▸), (CuI)(2,3-di­methyl­pyrazine) (Refcode LIDXUS, Jess *et al.*, 2007*a* ▸) and (CuI)_2_(2,3-di­methyl­pyrazine) [Refcodes LIDYAZ (Jess *et al.*, 2007*a* ▸) and LIDYAZ01 (Xu *et al.*, 2020 ▸)].

## 5. Synthesis and crystallization

**Synthesis**

CuCl and 2,3-di­methyl­pyrazine were purchased from Sigma-Aldrich.

**Figure 4:**
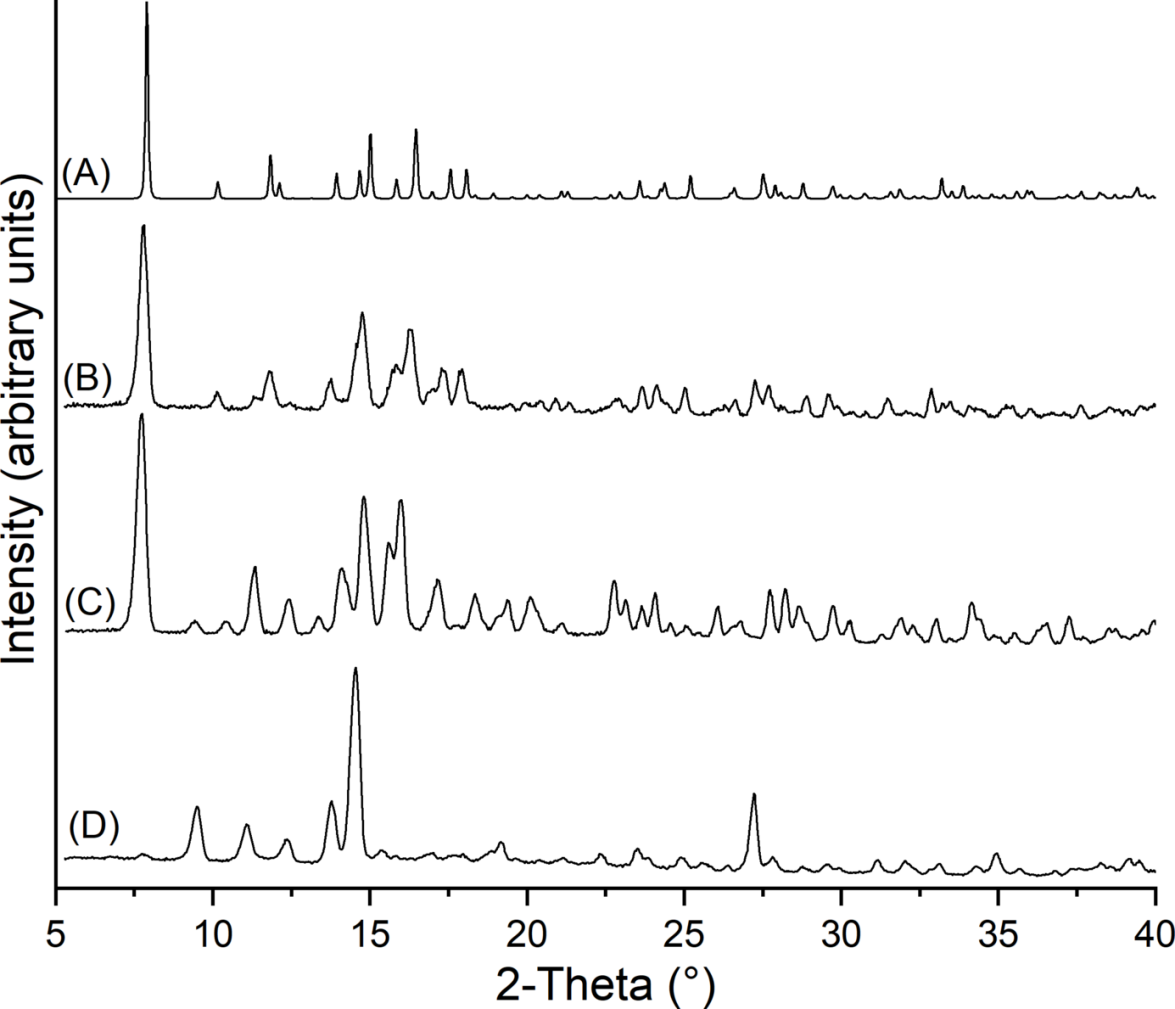
Calculated PXRD pattern of the title compound (A) and experimental powder pattern of a freshly prepared sample (B) and after storing this sample for 10 min (C) and for 24 h (D) at room temperature.

Light-orange single crystals were obtained within three days by the reaction of 99.0 mg (1 mmol) of CuCl and 108.14 mg (1 mmol) of 2,3-di­methyl­pyrazine) in 2 mL of ethanol. Larger amounts of a microcrystalline powder were obtained by stirring stoichiometric ratios of CuCl and 2,3-di­methyl­pyrazine in ethanol. Powder X-ray diffraction measurements proved that a pure crystalline phase had been obtained that is unstable and decomposes immediately into an unknown crystalline phase (Fig. 4 ▸).

**Experimental details**

The PXRD measurements were performed with Cu *K*α_1_ radiation (λ = 1.540598 Å) using a Stoe Transmission Powder Diffraction System (STADI P) equipped with a MYTHEN 1K detector and a Johansson-type Ge(111) monochromator.

## 6. Refinement

Crystal data, data collection and structure refinement details are summarized in Table 3 ▸. The C—H hydrogen atoms of the pyrazine rings and the ethanol mol­ecule were positioned with an idealized geometry and refined isotropically with *U*_iso_(H) = 1.2*U*_eq_(C) (1.5 for methyl H atoms). The methyl H atoms of the pyrazine rings are disordered and were positioned in two orientations rotated by 60° and were refined isotropically with *U*_iso_(H) = 1.5*U*_eq_(C). The O—H H atoms of the ethanol mol­ecules were located in difference maps, their bond lengths were set to ideal values and finally they were refined isotropically with *U*_iso_(H) = 1.5*U*_eq_(O). The ethanol solvate mol­ecule is disordered over two orientations and was refined with restraints (SAME, RIGU).

## Supplementary Material

Crystal structure: contains datablock(s) I. DOI: 10.1107/S2056989024009174/nx2014sup1.cif

Structure factors: contains datablock(s) I. DOI: 10.1107/S2056989024009174/nx2014Isup2.hkl

Figure S1. Crystal structure of the orthorhombic (top) and the monoclinic (bottom) polymorph of CuCl(2,3-dimethylpyrazine). The H atoms are omitted for clarity. DOI: 10.1107/S2056989024009174/nx2014sup3.png

CCDC reference: 2385254

Additional supporting information:  crystallographic information; 3D view; checkCIF report

## Figures and Tables

**Table 1:** Selected bond lengths (Å)

Cu1—Cu2	2.9516 (5)	Cu2—Cl1	2.4047 (8)
Cu1—Cl1	2.4055 (8)	Cu2—Cl2	2.4641 (9)
Cu1—Cl2	2.3768 (8)	Cu2—N2^i^	2.026 (2)
Cu1—N1	2.063 (2)	Cu2—N12^ii^	2.031 (2)
Cu1—N11	2.061 (2)		

Symmetry codes: (i) 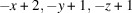; (ii) 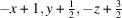.

**Table 2:** Hydrogen-bond geometry (Å, °)

*D*—H⋯*A*	*D*—H	H⋯*A*	*D*⋯*A*	*D*—H⋯*A*
C3—H3⋯Cl2^i^	0.95	2.79	3.463 (3)	128
C4—H4⋯Cl1	0.95	2.77	3.444 (3)	128
C4—H4⋯Cl1^iii^	0.95	2.81	3.513 (3)	132
C5—H5*B*⋯Cl1^iv^	0.98	2.83	3.609 (3)	137
C6—H6*C*⋯Cl1^iv^	0.98	2.98	3.653 (3)	127
C13—H13⋯Cl1^v^	0.95	2.75	3.395 (3)	126
C15—H15*A*⋯Cl2	0.98	2.85	3.805 (4)	166
C15—H15*B*⋯O1′^vi^	0.98	2.51	3.114 (10)	120
C15—H15*E*⋯Cl1	0.98	2.74	3.606 (4)	148
C16—H16*B*⋯O1	0.98	2.53	3.372 (11)	144
C16—H16*D*⋯O1′	0.98	2.41	3.275 (10)	147
O1—H1⋯Cl2^vii^	0.88	2.36	3.158 (7)	151

Symmetry codes: (i) 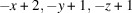; (iii) 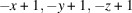; (iv) 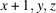; (v) 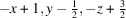; (vi) 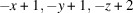; (vii) 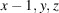.

**Table 3:** Experimental details

Crystal data	
Chemical formula	[CuCl(C_6_H_8_N_2_)]·0.5C_2_H_6_O
*M*_r_	230.17
Crystal system, space group	Monoclinic, *P*2_1_/*c*
Temperature (K)	170
*a*, *b*, *c* (Å)	7.0557 (5), 14.5923 (8), 17.4171 (13)
β (°)	92.253 (9)
*V* (Å^3^)	1791.9 (2)
*Z*	8
Radiation type	Mo *K*α
μ (mm^−1^)	2.68
Crystal size (mm)	0.4 × 0.3 × 0.25

Data collection	
Diffractometer	Stoe *IPDS2*
Absorption correction	Numerical (*X-SHAPE* and *X-RED* 32; Stoe, 2008 ▸)
*T*_min_, *T*_max_	0.603, 0.729
No. of measured, independent and observed [*I* > 2σ(*I*)] reflections	16726, 4230, 3278
*R*_int_	0.058
(sin θ/λ)_max_ (Å^−1^)	0.662

Refinement	
*R*[*F*^2^ > 2σ(*F*^2^)], *wR*(*F*^2^), *S*	0.038, 0.100, 1.01
No. of reflections	4230
No. of parameters	243
No. of restraints	21
H-atom treatment	H-atom parameters constrained
Δρ_max_, Δρ_min_ (e Å^−3^)	1.05, −0.67

Computer programs: *X-AREA* (Stoe, 2008 ▸), *SHELXT2014/4* (Sheldrick, 2015*a* ▸), *SHELXL2016/6* (Sheldrick, 2015*b* ▸), *DIAMOND* (Brandenburg, 1999 ▸), *XP* in *SHELXTL-PC* (Sheldrick, 2008 ▸) and *publCIF* (Westrip, 2010 ▸).

## supplementary crystallographic information

### Poly[[µ-chlorido-µ-(2,3-dimethylpyrazine)-copper(I)] ethanol hemisolvate] Crystal data

**Table:**

[CuCl(C_6_H_8_N_2_)]·0.5C_2_H_6_O	*F*(000) = 936
*M**_r_* = 230.17	*D*_x_ = 1.706 Mg m^−^^3^
Monoclinic, *P*2_1_/*c*	Mo *K*α radiation, λ = 0.71073 Å
*a* = 7.0557 (5) Å	Cell parameters from 4230 reflections
*b* = 14.5923 (8) Å	θ = 2.3–28.1°
*c* = 17.4171 (13) Å	µ = 2.68 mm^−^^1^
β = 92.253 (9)°	*T* = 170 K
*V* = 1791.9 (2) Å^3^	Block, orange
*Z* = 8	0.4 × 0.3 × 0.25 mm

### Poly[[µ-chlorido-µ-(2,3-dimethylpyrazine)-copper(I)] ethanol hemisolvate] Data collection

**Table:**

Stoe IPDS-2 diffractometer	3278 reflections with *I* > 2σ(*I*)
ω scans	*R*_int_ = 0.058
Absorption correction: numerical (X-Shape and X-Red 32; Stoe, 2008)	θ_max_ = 28.1°, θ_min_ = 2.3°
*T*_min_ = 0.603, *T*_max_ = 0.729	*h* = −8→9
16726 measured reflections	*k* = −17→19
4230 independent reflections	*l* = −22→22

### Poly[[µ-chlorido-µ-(2,3-dimethylpyrazine)-copper(I)] ethanol hemisolvate] Refinement

**Table:**

Refinement on *F*^2^	Hydrogen site location: mixed
Least-squares matrix: full	H-atom parameters constrained
*R*[*F*^2^ > 2σ(*F*^2^)] = 0.038	*w* = 1/[σ^2^(*F*_o_^2^) + (0.0621*P*)^2^ + 0.265*P*] where *P* = (*F*_o_^2^ + 2*F*_c_^2^)/3
*wR*(*F*^2^) = 0.100	(Δ/σ)_max_ = 0.001
*S* = 1.01	Δρ_max_ = 1.05 e Å^−^^3^
4230 reflections	Δρ_min_ = −0.67 e Å^−^^3^
243 parameters	Extinction correction: *SHELXL2016/6* (Sheldrick, 2015b), Fc^*^=kFc[1+0.001xFc^2^λ^3^/sin(2θ)]^-1/4^
21 restraints	Extinction coefficient: 0.0066 (9)

### Poly[[µ-chlorido-µ-(2,3-dimethylpyrazine)-copper(I)] ethanol hemisolvate] Special details

**Table:**

Geometry. All esds (except the esd in the dihedral angle between two l.s. planes) are estimated using the full covariance matrix. The cell esds are taken into account individually in the estimation of esds in distances, angles and torsion angles; correlations between esds in cell parameters are only used when they are defined by crystal symmetry. An approximate (isotropic) treatment of cell esds is used for estimating esds involving l.s. planes.

### Poly[[µ-chlorido-µ-(2,3-dimethylpyrazine)-copper(I)] ethanol hemisolvate] Fractional atomic coordinates and isotropic or equivalent isotropic displacement parameters (Å^2^)

**Table:**

	*x*	*y*	*z*	*U*_iso_*/*U*_eq_	Occ. (<1)
Cu1	0.78188 (6)	0.47540 (2)	0.68448 (2)	0.01808 (12)	
Cu2	0.71798 (6)	0.66110 (3)	0.62144 (2)	0.02162 (13)	
Cl1	0.50112 (10)	0.53390 (5)	0.61872 (4)	0.01799 (16)	
Cl2	0.93604 (11)	0.61192 (5)	0.72751 (4)	0.02134 (17)	
N1	0.9212 (4)	0.41035 (16)	0.59795 (13)	0.0137 (5)	
C1	1.0935 (4)	0.37340 (19)	0.60741 (17)	0.0146 (5)	
C2	1.1961 (4)	0.34341 (19)	0.54407 (17)	0.0152 (5)	
N2	1.1223 (4)	0.35171 (16)	0.47210 (14)	0.0145 (5)	
C3	0.9452 (4)	0.38599 (19)	0.46354 (16)	0.0151 (5)	
H3	0.887739	0.390104	0.413414	0.018*	
C4	0.8454 (4)	0.41518 (19)	0.52532 (16)	0.0151 (5)	
H4	0.721120	0.439149	0.516909	0.018*	
C5	1.1757 (5)	0.3646 (2)	0.68748 (17)	0.0214 (6)	
H5A	1.096504	0.398317	0.722833	0.032*	0.5
H5B	1.304413	0.389971	0.689939	0.032*	0.5
H5C	1.180087	0.299793	0.702159	0.032*	0.5
H5D	1.290832	0.327071	0.687121	0.032*	0.5
H5E	1.082923	0.335416	0.720015	0.032*	0.5
H5F	1.207250	0.425594	0.707795	0.032*	0.5
C6	1.3878 (5)	0.3008 (2)	0.55575 (19)	0.0222 (6)	
H6A	1.457658	0.306373	0.508440	0.033*	0.5
H6B	1.373623	0.235865	0.568711	0.033*	0.5
H6C	1.457764	0.332212	0.597738	0.033*	0.5
H6D	1.401705	0.276593	0.608153	0.033*	0.5
H6E	1.485740	0.347102	0.547881	0.033*	0.5
H6F	1.401599	0.250755	0.518855	0.033*	0.5
N11	0.6763 (4)	0.39165 (16)	0.76792 (14)	0.0157 (5)	
C11	0.5375 (4)	0.41740 (19)	0.81394 (16)	0.0157 (6)	
C12	0.4226 (4)	0.3518 (2)	0.84878 (16)	0.0170 (6)	
N12	0.4515 (4)	0.26194 (16)	0.83942 (14)	0.0164 (5)	
C13	0.5989 (5)	0.2370 (2)	0.79683 (18)	0.0191 (6)	
H13	0.628090	0.173794	0.791745	0.023*	
C14	0.7076 (5)	0.3006 (2)	0.76071 (18)	0.0206 (6)	
H14	0.807457	0.280241	0.729885	0.025*	
C15	0.5046 (6)	0.5175 (2)	0.8253 (2)	0.0280 (7)	
H15A	0.610176	0.552254	0.804847	0.042*	0.5
H15B	0.496102	0.530378	0.880324	0.042*	0.5
H15C	0.385951	0.535553	0.798348	0.042*	0.5
H15D	0.384643	0.526536	0.850832	0.042*	0.5
H15E	0.498718	0.548412	0.775355	0.042*	0.5
H15F	0.608868	0.543238	0.857332	0.042*	0.5
C16	0.2612 (6)	0.3801 (2)	0.8967 (2)	0.0345 (9)	
H16A	0.167282	0.330600	0.897336	0.052*	0.5
H16B	0.201857	0.435436	0.874824	0.052*	0.5
H16C	0.308577	0.392655	0.949267	0.052*	0.5
H16D	0.284528	0.441861	0.916949	0.052*	0.5
H16E	0.249954	0.337025	0.939461	0.052*	0.5
H16F	0.143234	0.379806	0.865018	0.052*	0.5
O1	−0.0035 (15)	0.5719 (6)	0.9050 (5)	0.085 (3)	0.549 (9)
H1	−0.064317	0.583978	0.861134	0.127*	0.549 (9)
C21	0.1135 (13)	0.6449 (7)	0.9304 (6)	0.0394 (19)	0.549 (9)
H21A	0.036154	0.701227	0.933706	0.047*	0.549 (9)
H21B	0.211718	0.655766	0.892405	0.047*	0.549 (9)
C22	0.209 (3)	0.6259 (12)	1.0078 (9)	0.048 (3)	0.549 (9)
H22A	0.127327	0.586258	1.037764	0.072*	0.549 (9)
H22B	0.230508	0.683818	1.035287	0.072*	0.549 (9)
H22C	0.330546	0.595308	1.000773	0.072*	0.549 (9)
O1'	0.2744 (15)	0.5440 (6)	1.0252 (5)	0.067 (3)	0.451 (9)
H1'	0.188987	0.511696	1.043788	0.101*	0.451 (9)
C21'	0.200 (3)	0.6295 (13)	1.0044 (8)	0.043 (2)	0.451 (9)
H21C	0.307017	0.669614	0.991192	0.052*	0.451 (9)
H21D	0.142396	0.656268	1.050232	0.052*	0.451 (9)
C22'	0.054 (2)	0.6334 (9)	0.9387 (7)	0.045 (2)	0.451 (9)
H22D	0.104732	0.668114	0.895999	0.068*	0.451 (9)
H22E	−0.061256	0.663527	0.955912	0.068*	0.451 (9)
H22F	0.022724	0.570994	0.921519	0.068*	0.451 (9)

### Poly[[µ-chlorido-µ-(2,3-dimethylpyrazine)-copper(I)] ethanol hemisolvate] Atomic displacement parameters (Å^2^)

**Table:**

	*U*^11^	*U*^22^	*U*^33^	*U*^12^	*U*^13^	*U*^23^
Cu1	0.0204 (2)	0.0183 (2)	0.01597 (19)	−0.00030 (14)	0.00639 (14)	0.00095 (13)
Cu2	0.0211 (2)	0.0198 (2)	0.0248 (2)	0.00221 (14)	0.01132 (15)	−0.00322 (14)
Cl1	0.0147 (4)	0.0174 (3)	0.0219 (3)	0.0010 (2)	0.0017 (2)	−0.0021 (2)
Cl2	0.0239 (4)	0.0218 (4)	0.0183 (3)	−0.0067 (3)	0.0009 (3)	−0.0019 (3)
N1	0.0137 (13)	0.0139 (11)	0.0136 (11)	−0.0018 (8)	0.0026 (9)	−0.0003 (8)
C1	0.0142 (15)	0.0123 (12)	0.0174 (13)	−0.0030 (10)	0.0012 (10)	0.0020 (10)
C2	0.0118 (14)	0.0115 (12)	0.0225 (14)	0.0007 (10)	0.0027 (10)	0.0009 (10)
N2	0.0151 (13)	0.0115 (11)	0.0173 (11)	0.0001 (8)	0.0042 (9)	0.0000 (9)
C3	0.0141 (15)	0.0158 (13)	0.0155 (13)	−0.0015 (10)	0.0025 (10)	0.0007 (10)
C4	0.0129 (15)	0.0150 (13)	0.0176 (13)	0.0009 (10)	0.0025 (10)	0.0012 (10)
C5	0.0191 (17)	0.0283 (16)	0.0168 (14)	0.0002 (12)	0.0003 (11)	0.0031 (11)
C6	0.0147 (16)	0.0234 (16)	0.0289 (16)	0.0070 (11)	0.0044 (12)	0.0014 (12)
N11	0.0160 (13)	0.0163 (12)	0.0153 (11)	0.0003 (9)	0.0054 (9)	0.0026 (9)
C11	0.0182 (16)	0.0155 (13)	0.0135 (13)	0.0009 (10)	0.0032 (10)	0.0011 (10)
C12	0.0187 (16)	0.0180 (14)	0.0149 (13)	0.0009 (11)	0.0070 (11)	0.0021 (10)
N12	0.0167 (13)	0.0148 (11)	0.0181 (12)	−0.0013 (9)	0.0054 (9)	0.0029 (9)
C13	0.0202 (16)	0.0142 (13)	0.0235 (14)	0.0002 (11)	0.0088 (11)	0.0001 (11)
C14	0.0216 (17)	0.0154 (14)	0.0256 (15)	0.0021 (11)	0.0114 (12)	−0.0002 (11)
C15	0.040 (2)	0.0151 (14)	0.0301 (17)	0.0030 (13)	0.0179 (15)	0.0020 (12)
C16	0.037 (2)	0.0229 (17)	0.046 (2)	0.0030 (14)	0.0295 (18)	0.0040 (15)
O1	0.114 (5)	0.078 (4)	0.059 (4)	−0.032 (4)	−0.040 (4)	0.022 (3)
C21	0.027 (4)	0.048 (3)	0.043 (3)	0.019 (2)	0.000 (3)	−0.002 (2)
C22	0.052 (6)	0.044 (4)	0.048 (3)	0.016 (3)	−0.012 (4)	−0.002 (3)
O1'	0.078 (5)	0.050 (3)	0.074 (5)	−0.006 (3)	−0.007 (4)	−0.002 (3)
C21'	0.052 (5)	0.049 (3)	0.030 (3)	−0.007 (3)	0.029 (3)	0.001 (3)
C22'	0.057 (5)	0.049 (4)	0.032 (3)	0.007 (4)	0.026 (3)	−0.013 (3)

### Poly[[µ-chlorido-µ-(2,3-dimethylpyrazine)-copper(I)] ethanol hemisolvate] Geometric parameters (Å, º)

**Table:**

Cu1—Cu2	2.9516 (5)	C12—N12	1.338 (4)
Cu1—Cl1	2.4055 (8)	C12—C16	1.496 (4)
Cu1—Cl2	2.3768 (8)	N12—C13	1.351 (4)
Cu1—N1	2.063 (2)	C13—H13	0.9500
Cu1—N11	2.061 (2)	C13—C14	1.373 (4)
Cu2—Cl1	2.4047 (8)	C14—H14	0.9500
Cu2—Cl2	2.4641 (9)	C15—H15A	0.9800
Cu2—N2^i^	2.026 (2)	C15—H15B	0.9800
Cu2—N12^ii^	2.031 (2)	C15—H15C	0.9800
N1—C1	1.334 (4)	C15—H15D	0.9800
N1—C4	1.356 (4)	C15—H15E	0.9800
C1—C2	1.412 (4)	C15—H15F	0.9800
C1—C5	1.495 (4)	C16—H16A	0.9800
C2—N2	1.344 (4)	C16—H16B	0.9800
C2—C6	1.496 (4)	C16—H16C	0.9800
N2—C3	1.349 (4)	C16—H16D	0.9800
C3—H3	0.9500	C16—H16E	0.9800
C3—C4	1.377 (4)	C16—H16F	0.9800
C4—H4	0.9500	O1—H1	0.8792
C5—H5A	0.9800	O1—C21	1.408 (13)
C5—H5B	0.9800	C21—H21A	0.9900
C5—H5C	0.9800	C21—H21B	0.9900
C5—H5D	0.9800	C21—C22	1.508 (11)
C5—H5E	0.9800	C22—H22A	0.9800
C5—H5F	0.9800	C22—H22B	0.9800
C6—H6A	0.9800	C22—H22C	0.9800
C6—H6B	0.9800	O1'—H1'	0.8401
C6—H6C	0.9800	O1'—C21'	1.396 (18)
C6—H6D	0.9800	C21'—H21C	0.9900
C6—H6E	0.9800	C21'—H21D	0.9900
C6—H6F	0.9800	C21'—C22'	1.512 (14)
N11—C11	1.343 (4)	C22'—H22D	0.9800
N11—C14	1.353 (4)	C22'—H22E	0.9800
C11—C12	1.407 (4)	C22'—H22F	0.9800
C11—C15	1.494 (4)		
			
Cl1—Cu1—Cu2	52.14 (2)	C14—N11—Cu1	116.86 (19)
Cl2—Cu1—Cu2	53.79 (2)	N11—C11—C12	120.9 (3)
Cl2—Cu1—Cl1	102.17 (3)	N11—C11—C15	118.3 (3)
N1—Cu1—Cu2	102.76 (7)	C12—C11—C15	120.8 (3)
N1—Cu1—Cl1	102.87 (7)	C11—C12—C16	121.1 (3)
N1—Cu1—Cl2	113.00 (7)	N12—C12—C11	121.4 (3)
N11—Cu1—Cu2	138.82 (7)	N12—C12—C16	117.4 (3)
N11—Cu1—Cl1	103.44 (8)	C12—N12—Cu2^iii^	125.1 (2)
N11—Cu1—Cl2	116.64 (7)	C12—N12—C13	117.1 (2)
N11—Cu1—N1	115.99 (10)	C13—N12—Cu2^iii^	117.7 (2)
Cl1—Cu2—Cu1	52.16 (2)	N12—C13—H13	119.1
Cl1—Cu2—Cl2	99.68 (3)	N12—C13—C14	121.7 (3)
Cl2—Cu2—Cu1	51.10 (2)	C14—C13—H13	119.1
N2^i^—Cu2—Cu1	97.63 (7)	N11—C14—C13	121.7 (3)
N2^i^—Cu2—Cl1	106.65 (7)	N11—C14—H14	119.2
N2^i^—Cu2—Cl2	103.03 (8)	C13—C14—H14	119.2
N2^i^—Cu2—N12^ii^	133.66 (10)	C11—C15—H15A	109.5
N12^ii^—Cu2—Cu1	128.59 (7)	C11—C15—H15B	109.5
N12^ii^—Cu2—Cl1	100.57 (8)	C11—C15—H15C	109.5
N12^ii^—Cu2—Cl2	108.53 (8)	C11—C15—H15D	109.5
Cu2—Cl1—Cu1	75.70 (3)	C11—C15—H15E	109.5
Cu1—Cl2—Cu2	75.11 (3)	C11—C15—H15F	109.5
C1—N1—Cu1	123.63 (19)	H15A—C15—H15B	109.5
C1—N1—C4	117.4 (2)	H15A—C15—H15C	109.5
C4—N1—Cu1	118.4 (2)	H15A—C15—H15D	141.1
N1—C1—C2	121.4 (3)	H15A—C15—H15E	56.3
N1—C1—C5	117.9 (3)	H15A—C15—H15F	56.3
C2—C1—C5	120.7 (3)	H15B—C15—H15C	109.5
C1—C2—C6	120.7 (3)	H15B—C15—H15D	56.3
N2—C2—C1	120.6 (3)	H15B—C15—H15E	141.1
N2—C2—C6	118.6 (3)	H15B—C15—H15F	56.3
C2—N2—Cu2^i^	122.3 (2)	H15C—C15—H15D	56.3
C2—N2—C3	117.4 (2)	H15C—C15—H15E	56.3
C3—N2—Cu2^i^	119.17 (19)	H15C—C15—H15F	141.1
N2—C3—H3	119.0	H15D—C15—H15E	109.5
N2—C3—C4	121.9 (3)	H15D—C15—H15F	109.5
C4—C3—H3	119.0	H15E—C15—H15F	109.5
N1—C4—C3	121.2 (3)	C12—C16—H16A	109.5
N1—C4—H4	119.4	C12—C16—H16B	109.5
C3—C4—H4	119.4	C12—C16—H16C	109.5
C1—C5—H5A	109.5	C12—C16—H16D	109.5
C1—C5—H5B	109.5	C12—C16—H16E	109.5
C1—C5—H5C	109.5	C12—C16—H16F	109.5
C1—C5—H5D	109.5	H16A—C16—H16B	109.5
C1—C5—H5E	109.5	H16A—C16—H16C	109.5
C1—C5—H5F	109.5	H16A—C16—H16D	141.1
H5A—C5—H5B	109.5	H16A—C16—H16E	56.3
H5A—C5—H5C	109.5	H16A—C16—H16F	56.3
H5A—C5—H5D	141.1	H16B—C16—H16C	109.5
H5A—C5—H5E	56.3	H16B—C16—H16D	56.3
H5A—C5—H5F	56.3	H16B—C16—H16E	141.1
H5B—C5—H5C	109.5	H16B—C16—H16F	56.3
H5B—C5—H5D	56.3	H16C—C16—H16D	56.3
H5B—C5—H5E	141.1	H16C—C16—H16E	56.3
H5B—C5—H5F	56.3	H16C—C16—H16F	141.1
H5C—C5—H5D	56.3	H16D—C16—H16E	109.5
H5C—C5—H5E	56.3	H16D—C16—H16F	109.5
H5C—C5—H5F	141.1	H16E—C16—H16F	109.5
H5D—C5—H5E	109.5	C21—O1—H1	112.5
H5D—C5—H5F	109.5	O1—C21—H21A	109.2
H5E—C5—H5F	109.5	O1—C21—H21B	109.2
C2—C6—H6A	109.5	O1—C21—C22	112.2 (12)
C2—C6—H6B	109.5	H21A—C21—H21B	107.9
C2—C6—H6C	109.5	C22—C21—H21A	109.2
C2—C6—H6D	109.5	C22—C21—H21B	109.2
C2—C6—H6E	109.5	C21—C22—H22A	109.5
C2—C6—H6F	109.5	C21—C22—H22B	109.5
H6A—C6—H6B	109.5	C21—C22—H22C	109.5
H6A—C6—H6C	109.5	H22A—C22—H22B	109.5
H6A—C6—H6D	141.1	H22A—C22—H22C	109.5
H6A—C6—H6E	56.3	H22B—C22—H22C	109.5
H6A—C6—H6F	56.3	C21'—O1'—H1'	109.4
H6B—C6—H6C	109.5	O1'—C21'—H21C	107.8
H6B—C6—H6D	56.3	O1'—C21'—H21D	107.8
H6B—C6—H6E	141.1	O1'—C21'—C22'	117.9 (16)
H6B—C6—H6F	56.3	H21C—C21'—H21D	107.2
H6C—C6—H6D	56.3	C22'—C21'—H21C	107.8
H6C—C6—H6E	56.3	C22'—C21'—H21D	107.8
H6C—C6—H6F	141.1	C21'—C22'—H22D	109.5
H6D—C6—H6E	109.5	C21'—C22'—H22E	109.5
H6D—C6—H6F	109.5	C21'—C22'—H22F	109.5
H6E—C6—H6F	109.5	H22D—C22'—H22E	109.5
C11—N11—Cu1	123.18 (19)	H22D—C22'—H22F	109.5
C11—N11—C14	117.1 (2)	H22E—C22'—H22F	109.5
			
Cu1—N1—C1—C2	−169.2 (2)	C5—C1—C2—N2	−179.7 (3)
Cu1—N1—C1—C5	10.7 (4)	C5—C1—C2—C6	1.0 (4)
Cu1—N1—C4—C3	169.7 (2)	C6—C2—N2—Cu2^i^	−15.6 (4)
Cu1—N11—C11—C12	156.2 (2)	C6—C2—N2—C3	176.6 (3)
Cu1—N11—C11—C15	−22.1 (4)	N11—C11—C12—N12	2.2 (5)
Cu1—N11—C14—C13	−159.5 (3)	N11—C11—C12—C16	−176.7 (3)
Cu2^i^—N2—C3—C4	−165.5 (2)	C11—N11—C14—C13	2.0 (5)
Cu2^iii^—N12—C13—C14	172.4 (3)	C11—C12—N12—Cu2^iii^	−174.2 (2)
N1—C1—C2—N2	0.2 (4)	C11—C12—N12—C13	1.8 (4)
N1—C1—C2—C6	−179.0 (3)	C12—N12—C13—C14	−3.9 (5)
C1—N1—C4—C3	−2.2 (4)	N12—C13—C14—N11	2.1 (5)
C1—C2—N2—Cu2^i^	165.1 (2)	C14—N11—C11—C12	−4.1 (4)
C1—C2—N2—C3	−2.7 (4)	C14—N11—C11—C15	177.7 (3)
C2—N2—C3—C4	2.7 (4)	C15—C11—C12—N12	−179.5 (3)
N2—C3—C4—N1	−0.2 (4)	C15—C11—C12—C16	1.5 (5)
C4—N1—C1—C2	2.2 (4)	C16—C12—N12—Cu2^iii^	4.7 (4)
C4—N1—C1—C5	−177.8 (2)	C16—C12—N12—C13	−179.2 (3)

Symmetry codes: (i) −*x*+2, −*y*+1, −*z*+1; (ii) −*x*+1, *y*+1/2, −*z*+3/2; (iii) −*x*+1, *y*−1/2, −*z*+3/2.

### Poly[[µ-chlorido-µ-(2,3-dimethylpyrazine)-copper(I)] ethanol hemisolvate] Hydrogen-bond geometry (Å, º)

**Table:**

*D*—H···*A*	*D*—H	H···*A*	*D*···*A*	*D*—H···*A*
C3—H3···Cl2^i^	0.95	2.79	3.463 (3)	128
C4—H4···Cl1	0.95	2.77	3.444 (3)	128
C4—H4···Cl1^iv^	0.95	2.81	3.513 (3)	132
C5—H5*B*···Cl1^v^	0.98	2.83	3.609 (3)	137
C6—H6*C*···Cl1^v^	0.98	2.98	3.653 (3)	127
C13—H13···Cl1^iii^	0.95	2.75	3.395 (3)	126
C15—H15*A*···Cl2	0.98	2.85	3.805 (4)	166
C15—H15*B*···O1′^vi^	0.98	2.51	3.114 (10)	120
C15—H15*E*···Cl1	0.98	2.74	3.606 (4)	148
C16—H16*B*···O1	0.98	2.53	3.372 (11)	144
C16—H16*D*···O1′	0.98	2.41	3.275 (10)	147
O1—H1···Cl2^vii^	0.88	2.36	3.158 (7)	151

Symmetry codes: (i) −*x*+2, −*y*+1, −*z*+1; (iii) −*x*+1, *y*−1/2, −*z*+3/2; (iv) −*x*+1, −*y*+1, −*z*+1; (v) *x*+1, *y*, *z*; (vi) −*x*+1, −*y*+1, −*z*+2; (vii) *x*−1, *y*, *z*.
