# Supplementary figures and images for: Synthesis and crystal structure of poly[[μ-chlorido-μ-(2,3-di­methyl­pyrazine)-copper(I)] ethanol hemisolvate], which shows a new isomeric CuCl(2,3-di­methyl­pyrazine) network
Source: Acta Crystallogr E Crystallogr Commun. 2024 Sep 24;80(Pt 10):1059–63. doi: 10.1107/S2056989024009174 (PMC11451487; doi:10.1107/S2056989024009174)

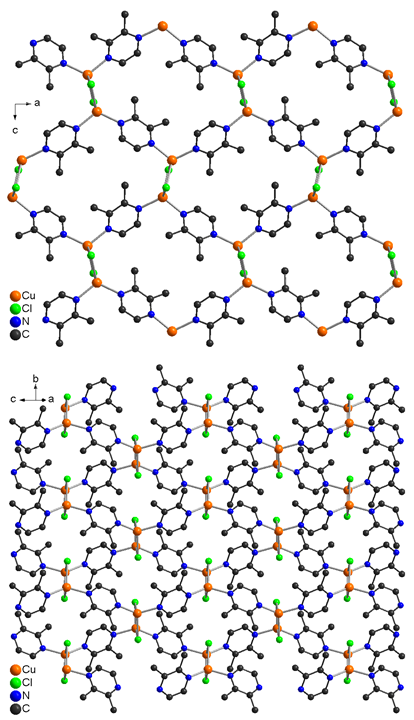

Supplement: Supplementary file 3 [file e-80-01059-sup3.png]
